# The effect of the modifiable areal unit problem on ecological model inference: A graphical simulation study for disease mapping in Australia

**DOI:** 10.1371/journal.pone.0329862

**Published:** 2025-12-18

**Authors:** James Hogg, Aiden Price, Conor Hassan, Shovanur Haque, Farzana Jahan, Wala Areed, Jessica Cameron, Susanna Cramb, Helen Thompson

**Affiliations:** 1 School of Mathematical Sciences, Queensland University of Technology (QUT), Brisbane, Queensland, Australia; 2 Australian Urban Research Infrastructure Network, University of Melbourne, Melbourne, Victoria, Australia; 3 Australian Centre for Health Services Innovation, School of Public Health and Social Work, QUT, Brisbane, Queensland, Australia; 4 School of Mathematics, Statistics, Chemistry and Physics, College of Science, Technology, Engineering and Mathematics, Murdoch University, Perth, Western Australia, Australia; 5 Descriptive Epidemiology, Cancer Council Queensland (CCQ), Brisbane, Queensland, Australia; Hainan University, CHINA

## Abstract

Statistical disease mapping is a valuable public health tool, as it identifies spatial patterns of disease occurrence. However, the Modifiable Areal Unit Problem (MAUP) poses challenges to disease mapping, as the aggregation of geographic units can impact statistical inferences. The effect of the MAUP depends on contextual factors, for example the geographic structure, aggregation level, choice of model, and the underlying data-generating process. We conducted a comprehensive simulation study to understand the role of these factors on the MAUP in the context of Australian disease mapping. We aggregated and rezoned disease count data at a fine geographic scale before fitting spatial and non-spatial regression models to assess the impact of the MAUP on coefficients. To aid the exploration of simulation results, we developed an interactive Shiny application that enables detailed and interactive exploration of the simulation results. This study highlights the need for disease mapping researchers to analyse sensitivity with rezoning and aggregation tools.

## Introduction

The rise of ecological analysis using methods from disease mapping is of increasing importance as data availability continues to improve [1]. Disease mapping generally provides spatially smoothed maps of disease outcomes across a study region [2], to aid policymakers in crafting targeted community-level interventions [3,4]. However, as highlighted by Tuson et al. [5], ecological analysis and disease maps that use data at a single spatial level (for example, postcode vs state) suffer from a variety of biases. The most notable is the modifiable areal unit problem (MAUP), which arises because of the significant dependence of statistical inference on the spatial configuration of the available data. Coined in 1979 by Openshaw [6], the MAUP has seen limited discussion in a majority of the ecological and disease mapping literature. For example, Tuson et al. [5] bring our attention to a review by Manley [7] who revealed that only 1% of papers using spatially aggregated data mentioned the MAUP. More recently, however, literature discussing the effect of the MAUP on disease mapping inference is increasing exponentially [8]. The recent interest in the MAUP is helping provide a solid platform for epidemiologists to consider the MAUP when conducting ecological analysis, thus reducing the bias of any inference or interventions based on them.

The MAUP is composed of two distinct but related sub-problems: the scale problem, which arises when inferences change as data are aggregated into larger or smaller spatial units [9], and the zoning problem, which refers to variation in results due to how boundaries are drawn, even when the number and size of units remain fixed. This distinction, originally articulated by Openshaw [6] and refined by Jelinski and Wu [10], is essential for understanding how spatial aggregation can influence statistical results. Our study explicitly examines both dimensions, using a simulation-based approach grounded in real-world geography to explore how aggregation level and boundary configuration affect model inference.

There are three main branches of research relating to the MAUP; what types of data are prone to the MAUP [11,12], strategies to avoid the MAUP [13] and how to present model results that acknowledge the issues of the MAUP [5,14]. Of course, the best solution to the MAUP is to avoid the use of aggregated data where possible [15]. However, where there are considerable ethical implications of analyzing and publishing results from individual-level data; the use of aggregated data is often the only feasible alternative.

Studies of the MAUP initially specify a minimal unit of data for inference. These minimal units could be individuals or aggregated data at a very high granularity. Investigation of the MAUP involves manipulating the construction and combination of the minimal units to assess changes in inference. In particular, one must consider two fundamental elements of the MAUP; scaling and zoning [16,17]. Scaling refers to changes in inference as the minimal units are aggregated into larger regions, whilst zoning refers to changes in inference from aggregating minimal units in different ways while maintaining the same average region size [18]. The effects of zoning can be investigated by “jittering” the boundaries of the aggregations, a technique used in many studies [14,16,17,19,20].

The MAUP can affect numerous aspects of the statistical analysis of aggregated data. A significant body of work has been carried out investigating the MAUP on means, variances and correlations [12,17,19]. In terms of model inference, scholars such as Briant et al. [21], Kok et al. [14] and Tuson et al. [16] have found significant variation in regression coefficients under different scaling and zoning. Given that aggregated spatial data is assumed to have some level of spatial autocorrelation, studies have also investigated the MAUP under varying degrees of spatial autocorrelation [15,17]. Early work by Fotheringham and Wong [15] found that spatial autocorrelation had little effect on regression coefficients from standard non-spatial linear models. The simulation study by Lee et al. [17] investigated the effect of the MAUP on means, variances and Moran’s I coefficients of rezoned and aggregated data for a wide range of spatial autocorrelation values at the minimal unit level. However, their work does not explore changes in model inference/coefficients.

In this work, we explore the impact of MAUP on the coefficient of an area level covariate using a simulation experiment based on Australian geography. Our work is novel in a variety of ways as we; use a real and unique geographic location for the simulations, compare results based on the rareness of the disease and the level of spatial autocorrelation, compare model inference with both spatial and non-spatial models and present a Shiny app to display the simulated data and results.

Unlike some studies, which simulate spatial grid data to investigate the MAUP [5], we use a real geographic area; Australia. According to the Australian Statistical Geography Standard (ASGS), the country can be split into a series of hierarchical statistical areas that completely cover Australia. Statistical Area Level 1 (SA1) is generally classed as the smallest level of the hierarchy, where SA2s are aggregations of SA1s and SA3s are aggregations of SA2s and so forth. Australia’s population is extremely decentralized – where about 80% of the population lives on the coast [22]. The Australian context makes for a very interesting study of the MAUP as the minimal units vary considerably in both population and geographic size. For example, some SA1s in outback Australia are geographically similar in size to greater capital cities, such as Sydney or Melbourne, which are themselves comprised of thousands of SA1s each.

We also investigate how the average number of counts for the minimal units can affect the MAUP. Aswi et al. [23] found that spatial models can accommodate spatial autocorrelation regardless of the number of counts. However, there is little literature on whether rare diseases are impacted differently by the MAUP compared with more common diseases. As highlighted by Lee et al. [17], there is a need to investigate how spatial autocorrelation affects ecological inference. The final aim of this work is to fit models to data with varying levels of spatial autocorrelation to assess the effect of the MAUP on regression coefficients and explore differences in model inference between spatial and non-spatial models.

## Materials and methods

In our simulation study, the minimal units are derived from the SA1s of the 2016 ASGS for New South Wales, Australian Capital Territory and Queensland. To ensure consistency during aggregation and simulation, we restricted the SA1s to only those that are completely geographically connected (that is, we excluded SA1s on islands), which resulted in M=30,418 SA1s. The populations for these SA1s were obatined from the 2016 Australian Census [24], where any area, *i*, with zero population, *N*_*i*_ = 0, was reset to *N*_*i*_ = 1 to ensure expected and observed counts could be simulated when applying the log function to *N*_*i*_.

### Simulating spatial data

Disease mapping often involves modelling observed counts that are related to the population size, an underlying rate of disease and some area-level covariates [2]. Given that we fixed the population sizes to SA1-level populations, we simulate both a single SA1-level covariate and the spatially correlated underlying rate of disease. The method of data simulation follows that of Morris et al. [25] and Aswi et al. [23].

To generate the rate of disease we simulate a spatially correlated random effect, $\phi \in {\mathbb{R}}^{M}$, using the proper conditional autoregressive (pCAR) prior. The spatial structure of the pCAR is governed by a symmetric binary contiguity matrix, $𝐖\in {\mathbb{R}}^{M\times M}$, where entry *W*_*ij*_ is equal to 1 if SA1 *i* and *j* are neighbours and zero otherwise. All diagonal terms of 𝐖 are zero. The pCAR is a relatively simple spatial prior to sample from given its multivariate Gaussian specification,


$$\phi \sim N\left(0,\sigma^{2}{\left(𝐃-\gamma 𝐖\right)}^{-1}\right),$$ (1)

where $𝐃=\text{diag}\left(\sum_{j}W_{1j},\ldots ,\sum_{j}W_{Mj}\right)$ and $\sigma^{2}$ is the random effect variance, which we set to 1 in this work. The parameter γ controls the level of spatial correlation in the random effects; with values close to 1 indicating high spatial autocorrelation in the random effects.

To construct the pCAR distribution, first calculate the sparse precision matrix as $\tau =𝐃\left(𝐈-\gamma {𝐃}^{-1}𝐖\right)$. Next, we take the Cholesky decomposition of $\tau ={𝐔}^{T}𝐔$, where 𝐔 is an upper triangular matrix and simulate a vector, $𝐳\in {\mathbb{R}}^{M}$, from a standard normal distribution, *N*(0,1). Finally, we solve 𝐔ϕ=𝐳 for the vector of spatially correlated random effects, $\phi \in {\mathbb{R}}^{M}$, using backward substitution. The single SA1-level continuous covariate, $𝐱\in {\mathbb{R}}^{M}$, for which inference changes are explored, is simulated from a Uniform(−3,3) distribution.

To generate the disease counts, we closely follow the steps outlined by Aswi et al. [23]. First, calculate the SA1-specific mean count for i=1,…,M, using $\mu_{i}=\text{exp}\left(\text{log}(N_{i})+\alpha +x_{i}+\phi_{i}\right)$. Next, we draw an initial vector of SA1-specific counts $y_{i}^{\text{initial}}\sim \text{Poisson}(\mu_{i})$ and then rescale ${𝐲}^{\text{initial}}=\left(y_{1}^{\text{initial}},\ldots ,y_{M}^{\text{initial}}\right)$ so it matches α, the desired average number of counts across all SA1s. This is achieved by $y_{i}=\text{round}\left(\alpha N\frac{y_{i}^{\text{initial}}}{\sum_{i=1}^{M}y_{i}^{\text{initial}}}\right)$. Following this, we can generate an initial vector of expected counts, $E_{i}^{\text{initial}}=N_{i}\frac{\sum_{i=1}^{M}y_{i}}{\sum_{i=1}^{M}N_{i}}$, which can be rescaled to ensure that $\sum_{i=1}^{M}y_{i}=\sum_{i=1}^{N}E_{i}$. The rescaled vector of expected counts, *E*_*i*_, is then used to calculate the raw incidence ratio (RIR) for each SA1, $RIR_{i}=\frac{y_{i}}{E_{i}}$.

The simulation parameters of interest are the rareness, represented by α, which is the average number of counts per SA1 across the simulated study region and γ, the level of spatial autocorrelation present in the random effects ϕ. In this work, we investigate the effect of MAUP with a combination of low, median and high counts (or rare to common) and spatial autocorrelation, which combine to form nine scenarios (see Table 1). The code to conduct the simulations can be found on GitHub [26].

**Table 1:** The nine data scenarios used to investigate the effects of the MAUP in our simulations.

		γ		
		0.05	0.5	0.95
α	5	S1	S2	S3
	25	S4	S5	S6
	50	S7	S8	S9

The three γ values were chosen as they are consistent with incidence rates for the most common cancers (e.g. keratinocyte cancers), all types of invasive cancers (excluding non-melanoma skin cancers) and female breast cancer for the low, median and high counts, respectively.

### Models

In this work, we are interested in the influence of aggregation on the association between the single covariate *x* and the disease counts, $𝐲=(y_{1},\ldots ,y_{M})$, which can be explored using a standard Poisson model of the form,


$$y_{i}\sim \text{Poisson}(\mu_{i})\;i=1,\ldots ,M$$ (2)

$$\text{log}\left(\mu_{i}\right)=\beta_{0}+\beta x_{i}+\psi \text{log}(E_{i})\;i=1,\ldots ,M$$

where *E*_*i*_ is the expected counts in area *i*, $\mu_{i}$ is the modeled log standardized incidence ratio (or log-relative risk), $\beta_{0}$ is an intercept term and β is the regression coefficient for *x* and the parameter of interest. The additional fixed effect ψ for $\text{log}(E_{i})$, is a relatively simple method to accommodate overdispersion in the data. The Poisson model in (2) is the baseline model from which we wish to empirically investigate whether point estimates for β vary when accommodating spatial autocorrelation.

To adjust for spatially correlated errors, we include a spatial random effect term, $v_{i}$, in the linear predictor of (2).


$$\mu_{i}=\beta_{0}+\beta x_{i}+\psi \text{log}(E_{i})+v_{i}\;\;\;\;\;i=1,\ldots ,M$$ (3)

We opt to use the popular spatial prior proposed by Leroux et al. [27] and further developed by MacNab [28]. The Leroux prior has several benefits over the common spatial BYM prior [29]. By employing only a single random effect that can capture both spatially structured and unstructured variation in the observed counts, the Leroux prior avoids significant identifiability issues of the BYM prior [30].


$$v_{i}|{𝐯}_{-i}\sim N\left(\frac{\rho \sum_{j=1}^{M}W_{ij}v_{j}}{1-\rho +\rho \sum_{j=1}^{M}W_{ij}},\frac{\tau^{2}}{1-\rho +\rho \sum_{j=1}^{M}W_{ij}}\right)$$ (4)

ρ~Uniform(0,1)

The inclusion of ρ ensures that the random effects, $𝐯=(v_{1},\ldots v_{N})$, are simultaneously smoothed toward the local and the global means. Depending on ρ, the Leroux prior can capture a wide range of variation. For example, if ρ=1, the prior captures variation equivalent to an intrinsic CAR prior [27]. On the other hand, when ρ=0, the Leroux prior captures variation equivalent to an independent random effect. The natural interplay of both spatially structured and unstructured random effects makes the Leroux prior a favourable option for disease mapping [2]. The parameter ρ is the relative contribution of the variance that is spatially structured as opposed to unstructured.

Throughout this work, we will refer to the generic non-spatial Poisson model (2) as the baseline model and the Poisson model with the Leroux spatial random effect (3) as the Leroux model. The Leroux model was fit using fully Bayesian inference with the CARBayes package in R (versions 5.2.4 and 4.0.5, respectively). We used 3 chains, 10,000 burn-in, followed by 40,000 iterations [31]. To reduce autocorrelation in the iterations from the component-wise Markov Chain Monte Carlo (MCMC) algorithm, we thinned the iterations by only keeping every 10th iteration. Convergence is a crucial aspect to be evaluated for any Bayesian model and can be done through a combination of diagnostic measures and visual checks. However, for this simulation study, where a large number of models were fitted, we chose to investigate the average $\hat{R}$ and effective sample size (ESS) across simulations [32]. For the covariate effect, β, all $\hat{R}<1.10$. In addition, the median $\hat{R}$ and ESS for β was 1.00 and 6255, respectively. The point estimates for β were the posterior median of the 12000 post-thinned iterations and the maximum likelihood estimates for the Leroux and baseline models, respectively.

### Zonations

A vital element of our simulation study is re-aggregation or zonation. Zonation is the process by which contiguous sub-areas are combined to form new and larger areas. Similar to Tuson et al. [16], who used SA1s in Perth, we used SA1 regions across Queensland (QLD), New South Wales (NSW), and the Australian Captial Territory (ACT) to generate unique zonations at each of seven levels of aggregation, t=1,…,7, each with increasing target populations, as shown in Table 2. The aggregation levels were labelled according to how the target populations compared with the median population of the areas within the ASGS. For example, the median population for SA2s was 8,454 and the median population for SA3s was 58,000, hence, the aggregation level with a target population of 10,000 was denoted SA2.5.

**Table 2:** Target population sizes provided to AZTool. The table displays the median and inter-quartile range (IQR) of the new zone populations for each aggregation level across all 100 unique zonations. We also provide the median and IQR of the area populations for the ASGS boundaries.

t	Area	Target Population	Simulation Median (IQR Bands)		ASGS Median (IQR Bands)	
0	SA1				402	(298 - 492)
1	SA1.5	5,000	5,948	(5,680 - 6,253)		
2	SA2	10,000	11,746	(11,207 - 12,429)	8,454	(5,004 - 14,228)
3	SA2.5	50,000	58,244	(54,512 - 63,238)		
4	SA3	90,000	104,998	(96,914 - 116,503)	57,857	(38,699 - 85,128)
5	SA3.5	200,000	233,698	(212,660 - 261,128)		
6	SA4	300,000	348,206	(314,588 - 397,207)	227,685	(189,294 - 314,397)
7	SA4.5	700,000	832,376	(748,992 - 936,821)		

**Fig 1:**
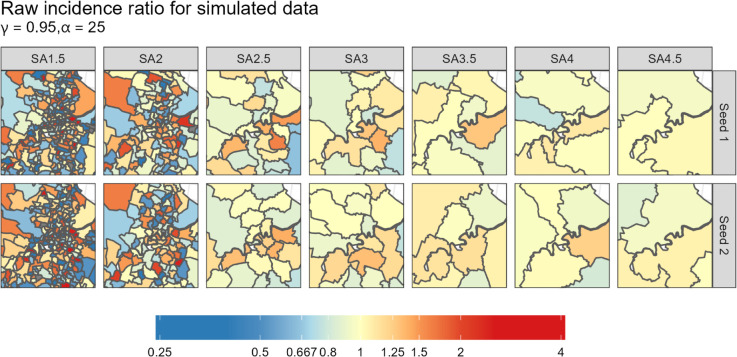
Map of Brisbane, Australia, which presents the RIRs for each of the seven aggregation levels (presented vertically) and 2 different zonation seeds (presented horizontally) for scenario 6.

Various zonation tools are available, including SKATER [33] and HeLP [34]. Zonation tools automate the creation of new aggregations for a given target population. In this work, we used AZTool [35], which has been used in a variety of studies of the MAUP [14,16,19,20]. For each of the seven target populations in Table 2 we created 100 unique zonations by providing AZTool with different starting seed values. See Fig 1 for two unique zonations generated using the same underlying simulated dataset. In addition to specifying a target population, AZTool accepts a minimum and maximum population threshold for the new zones. Following the recommendations by Tuson et al. [16], we take the minimum threshold to be 90% of the target population and set no maximum threshold. Hence, simulation medians and IQRs tended to be higher than the set target threshold. In addition to creating new zonations, we also aggregated and collected model results according to the ASGS boundaries of SA2, SA3 and SA4 which are similar to target populations, t=2,4,6.

For each of the nine simulation scenarios, we generate a single SA1 dataset using the method described in the *Simulating Spatial Data* Section. Using these datasets, we aggregate the SA1 data according to the zonations provided by AZTool. For each of the nine simulation scenarios, seven target populations, 100 seeds and two models, we collect the point estimates of β. Thus, the simulation study gives 9×7×100=6,300 randomly zoned and 9×3=27 officially zoned sets of data to which both the baseline and Leroux models were fit. The 100 point estimates for each scenario, target population, and model, provide an approximation to the zoning distribution of β. Burden and Steel [20] outline the theoretical properties of zoning distributions.

### Aggregation

For each target population AZTool assigns each SA1 to one of a series of new zones, $z_{t}=1,\ldots ,Z_{t}$, where *Z*_*t*_ is the total number of new zones at aggregation level *t*. For each zone and target population, the indices for SA1s in zone *z*_*t*_ are in the set $s_{zt}\in (s_{1},\ldots ,s_{n_{zt}})$, where *n*_*zt*_ is the number of SA1s assigned to zone *z*_*t*_ under target population *t*. The new zones are assigned the following values.


$$y_{zt}=\sum_{i\in s_{zt}}y_{i},\;x_{zt}=\frac{1}{n_{zt}}\sum_{i\in s_{zt}}x_{i}$$

$$E_{zt}=\sum_{i\in s_{zt}}E_{i},\;N_{zt}=\sum_{i\in s_{zt}}N_{i}$$

where $y_{zt},x_{zt},E_{zt}$ and *N*_*zt*_ are the total observed counts, mean covariate value, expected counts, and population size for zone *z* at target population *t*. These quantities are used to calculate $RIR_{zt}=\frac{y_{zt}}{E_{zt}}$. Note that all of these quantities can be further indexed by the AZTool seed number used. We also derive the Mean Absolute Error (MAE),


$${\text{MAE}}_{zt}=\frac{1}{n_{zt}}\sum_{i\in s_{zt}}\left|y_{i}-{\bar{y}}_{zt}\right|,$$ (5)

for each zonation and aggregation. The median MAE is displayed in the interactive application (see the *Results* Section). The MAE measures the sum of the absolute difference between the simulated counts of all the SA1s in the new zone compared to their average counts, ${\bar{y}}_{zt}=\frac{1}{n_{zt}}\sum_{i\in s_{zt}}y_{i}$.

## Results

**Fig 2:**
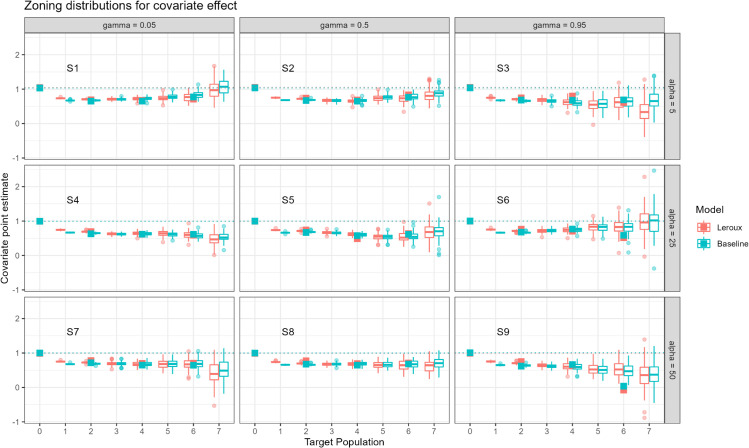
Plot displaying the zoning distribution for all scenarios, at all target population levels. The empty boxplots display the zoning distribution, whilst the filled boxes display the single point estimate derived by fitting the models to the official ABS ASGS zonation. As denoted in the legend, blue represents point estimates using the baseline (non-spatial) model, whilst red represents point estimates using the Leroux (spatial) model. Note that the true data generating process defines the covariate effect as 1 and that at, the simulation level (*t* = 0), both models produce β≈1. Note that these values overlap at *t* = 0, showing only the baseline model’s point estimate.

The primary output for this work is a set of simulation study results, where we considered model covariate effects under different scenarios. These scenarios applied rezoning and aggregation tools to generate 100 simulations at each aggregation level, then used to model data characterised by nine combinations of spatial autocorrelation and disease counts. Across all nine scenarios, Fig 2 indicates the increasing between-simulation variance of the zoning distributions for higher levels of aggregation. Low aggregation levels (t=1,2) display very small zoning variance but underestimate the true β=1. The bias induced by aggregating the data above the simulation level (*t* = 0), is consistent regardless of the spatial autocorrelation or rareness of the disease being modelling.

Fig 2 shows that as highly spatially autocorrelated (γ=0.95) data is aggregated, the zoning distributions approach the true parameter value of 1. This pattern is not observed for the other scenarios (low and mild levels of spatial autocorrelation), where both models consistently underestimate the underlying association. Finally, we observe that model inference using the ABS boundaries generally agrees with the zoning distributions, which supports their usefulness in practice.

**Fig 3:**
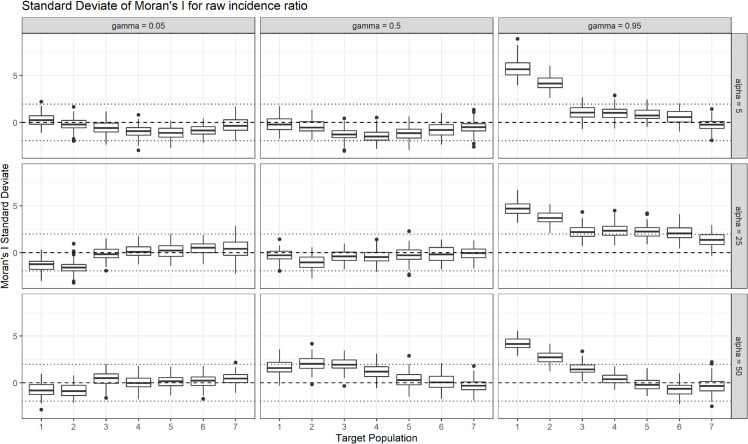
Plot displaying the Moran I standard deviate of the raw incidence ratio (RIR) across all scenarios and target populations. Each boxplot summarises the 100 Moran I values calculated on SA1 data aggregated according to each unique zonation. The small dotted lines on either side of zero (-1.96 and 1.96) represent the interval outside of which we would reject the null hypothesis of no spatial autocorrelation at the 0.05 level.

In addition to model estimates, for each simulation scenario, target population and zonation the Moran’s I statistics were also calculated [36]. Fig 3 follows the visualizations by Fotheringham and Wong [15] who presented the Moran’s I statistic vs the target population. Unlike these authors, we present the Moran’s I standard deviate, which is an equivalent measure but on the standard normal scale. The standard normal Moran’s I values allow us to compare results at different target populations. The plot shows the effect of aggregation on the global spatial autocorrelation of the RIRs. After aggregating highly spatially autocorrelated SA1 data (i.e. γ=0.95), Moran’s I becomes non-significant (the boxplots are within the interval) at the SA2.5 level.

### Interactive application

A novel element of this work was the development of a Shiny app to enable detailed and interactive exploration of the simulation results. The application, available at https://qutcds.shinyapps.io/MAUP_Analysis/ allows the user to alter aggregation, spatial autocorrelation (γ), and average counts (α) which are then displayed on a choropleth map. The displayed map is coloured according to the RIRs, with light yellows denoting RIRs lower than 1 and dark reds denoting RIRs higher than 1. In this work, we generated 100 unique zonations by setting the seed in AZTool. The user can randomly select a new seed for the simulated data and zonations to explore how unique zonations can be crafted with similar overall characteristics. The user can also select the official ASGS boundaries if these are available for the selected aggregation level.

The application includes an automated aggregation slider, allowing users to experience visually the impact of increasing the aggregation level with transitions on a single interactive map, expanding on the results shown in Fig 1. The reduction in the intensity of the RIRs is pronounced with the maps reducing to light yellows and reds as the aggregation level approaches *t* = 7.

The user can also access interactive versions of Fig 2 in the application. These interactive plots show the currently selected map values as large blue and red dots overlaid on the original plot. The Shiny app also includes similar boxplots for the intercept of the models. The interactive plots become very useful for larger aggregation levels, where changing the seed can have a drastic effect on the size of the coefficient for *x*.

We strongly urge readers to explore the Shiny app, as we believe it can help one gain an intuitive understanding of the MAUP. The app is particularly important in the Australian context as it enables the user to compare the official ASGS boundaries to other possible zonations.

## Discussion

This simulation study highlights several immediate findings relating to the general study of spatial data and spatial aggregation in practice. As expected, spatial data never gains spatial autocorrelation upon aggregation. Merging spatially-correlated regions leads to a decrease in variability and a reduction in the visibility of spatial trends. We observed decreased spatial autocorrelation across simulated data sets with low and moderate spatial autocorrelation, which immediately lost spatial dependence at the first aggregation step. This effect is mitigated in the case of moderate spatial autocorrelation (γ=0.5) paired with higher average counts (α=50), as increases in average incidence are directly tied to statistical power. Data with high spatial autocorrelation (γ=0.95) retain spatial dependence when aggregated up to a population target of 10,000 (target population 2, the ASGS equivalent of SA2) consistently, with medium average incidence counts (α=25) retaining spatial dependence up to a population target of 300,000 (target population 6, the ASGS equivalent of SA4).

These findings are integral to the study of the MAUP, given the ties between areal units, spatial aggregation, spatial autocorrelation, and model inference. Even considering areal unit changes with constant target populations, spatial aggregation is found in Fig 2 to immediately reduce or remove spatial dependence in simulation scenarios with low and moderate spatial autocorrelation. Figs 2 and 3 indicate that once aggregating to a target population of 90,000 or greater (target population 4, the ASGS equivalent of SA3), spatial autocorrelation across almost all of the scenarios decreases significantly, resulting in high model variance. This result supports the use of non-spatial models in Australian studies that use geographies larger than the SA2 resolution. Conversely, spatial models should always be considered for use in studies at the SA1 and SA2 resolution and, if non-spatial models are used, the effect of the MAUP should be acknowledged. In future, standards should be developed for sensitivity analysis with respect to region boundary rezoning to better capture sensitivity to the zoning sub-problem of the MAUP.

An additional noteworthy trend identified in Fig 2 is that regardless of average incidence counts, the variability in the coefficient point estimates across simulations increases as spatial autocorrelation increases, with this effect most readily apparent when comparing aggregation effects between γ=0.05 and γ=0.95. In the event of low spatial autocorrelation, random aggregation of nearby areas combines more heterogeneous areas, leading to higher information loss and less overall variability. On the other hand, in the event of high spatial autocorrelation, random aggregation of nearby areas combines more homogeneous areas, leading to lower information loss and more overall variability. As a result of this phenomenon, a modeller cannot feasibly determine whether the model variance is due to (a) the data, (b) the model, (c) the aggregation process or (d) the spatial autocorrelation of the underlying unit records. Hence, the MAUP is demonstrated. We do see, however, that this conundrum is minimal across all γ and α up to a population target of 10,000 (target population 2, the ASGS equivalent of SA2), suggesting a minimal impact of MAUP up to this level of aggregation.

This study has several limitations. While the Australian context offers valuable insights into the impact of the MAUP on heterogeneous population distributions, the effects of modified zonations, disease incidence, and spatial autocorrelation are likely to differ in regions with more uniform population distributions. Additionally, expanding the range of target populations in the simulation study and improving adherence to the model could provide a clearer understanding of how aggregation and spatial autocorrelation affect model inference. The value range of spatial autocorrelation and average counts used in the simulation study would enhance the robustness of the observed trends.

The limitations inherent to standard non-spatial and Bayesian spatial models, including assumptions about spatial structures and the treatment of spatial dependencies, may influence the findings of this study. Further research could explore the impact of the MAUP using a broader array of statistical models and methodologies. Additionally, methods to split up the variability of model results between data, model, aggregation process and spatial autocorrelation should be pursued to better understand the role of MAUP.

The simulation focused on count data, which may limit the applicability of the findings to other types of spatial data or different public health outcomes. The MAUP’s impact on continuous health outcomes, environmental data, or socio-economic indicators might differ and warrants further investigation.

The result of this simulation study is of particular interest due to rezonation techniques employed, as traditional spatial analysis assumes a fixed geographical structure. By varying the geographical structure, we capture variability resulting from aggregation and zonation. This study is internationally relevant despite the heterogeneous Australian landscape, as our analysis includes non-spatial and spatial models, with the latter’s spatial term based on region neighbours rather than the distance between regions. As such, this paper guides other countries on assessing MAUP for their geographies so that studies in those countries better understand when spatial models will add value to their analyses. This study also provides a starting point for investigating more complex issues surrounding the MAUP. For example, literature shows that spatial confounding impacts covariate inference when both the covariate and outcome are spatially correlated [37].

## Conclusion

To explore the MAUP in the Australian context, we simulated disease count data at fine-scale geographical resolution and examined the consequential effects of rezoning and aggregation on model inferences. In addition to displaying the results in this report, we developed an interactive Shiny app to allow the reader to explore the simulation results in more detail.

Our findings capture the impact of spatial aggregation and zonation on parameter estimates, considering differences in spatial autocorrelation. This study corroborates the existing MAUP literature, demonstrating that coefficient estimates’ variance significantly increases at greater aggregation levels. This study also contributes to the MAUP literature, demonstrating that the variance of the zoning distribution increases at greater aggregation levels. In the context of this study, the MAUP embodies the interdeterminancy introduced by spatial aggregation, where the observed variability in model outputs can be attributed to a confluence of factors: the intrinsic characteristics of count data, the spatial and non-spatial models employed, the methodological nuances of the aggregation process, and the existence or non-existence of spatial autocorrelation. These factors underscore the MAUP as not merely a statistical inconvenience but a substantive issue that necessitates robust methodological frameworks to discern the true drivers of observed spatial patterning.
